# Coverage-Dependent Hydrogen Adsorption, Spillover, and Vacancy Formation in Ni/ZrO_2_ Systems: A First-Principles DFT Investigation

**DOI:** 10.1021/acsomega.6c00781

**Published:** 2026-04-24

**Authors:** Eugenio F. Souza, Fábio S. Toniolo

**Affiliations:** 28125 Chemical Engineering Program of COPPE Federal University of Rio Janeiro (UFRJ), Rio de Janeiro CEP 21941-972, Brazil

## Abstract

Hydrogen spillover represents a fundamental mechanism in supported metal catalysts, yet its coverage-dependent behavior and relationship to oxygen vacancy formation remain not fully understood. In this line, the present theoretical investigation systematically examines the adsorption of hydrogen molecules (H_2_), the spillover of dissociated species (H*) and vacancy generation in a Ni/ZrO_2_ model system through varying hydrogen coverages. The results show that clean zirconia surfaces adsorb H_2_ weakly, showing a minor tendency for spontaneous bond cleavage. Conversely, in the presence of Ni, exothermic and spontaneous dissociative adsorption is observed. The results also reveal an interesting transformation in spillover energetics as a function of hydrogen coverage, transitioning from endothermic at low to exothermic at high coverages. This transformation coincides with a systematic decrease in oxygen vacancy formation energies, which is eventually facilitated by spillover. Structural analysis indicates that the Ni nanoparticle maintains stability up to some degree of hydrogen coverage before undergoing a more significant geometric reorganization at higher coverages. Electronic structure analysis indicates that spillover is governed by structural rearrangements rather than by charge transfer processes. Relationships were identified between hydrogen coverage and adsorption energies, spillover barriers, and vacancy formation energies, providing a quantitative framework under varying H_2_ conditions.

## Graphical abstract

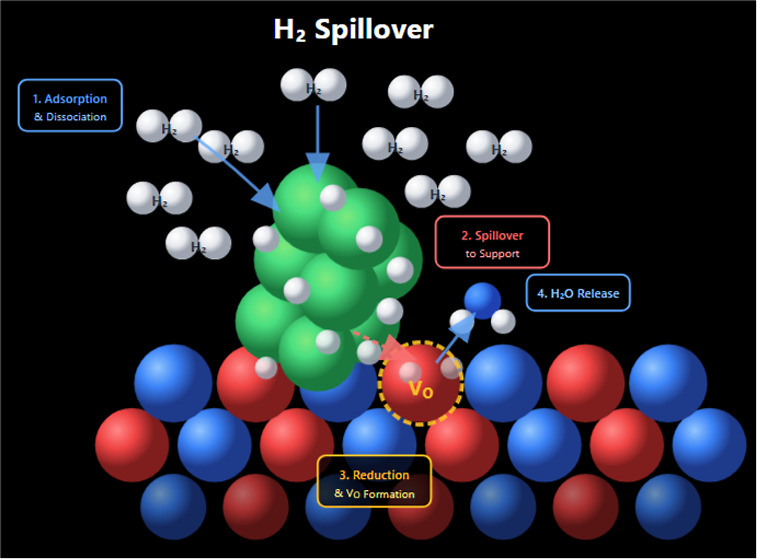

## Introduction

Experimental and theoretical advancements made in the past few years have significantly deepened the understanding of catalysts based on oxide-supported transition metals, particularly on low-dimensional systems like nanoparticles and single atoms.^1,2^ These catalysts are of great interest due to their tailorable properties, demonstrating enhanced activity and selectivity in crucial catalytic applications such as energy conversion and environmental remediation.^3–6^ While on one hand noble metals (e.g., Pt, Pd, Rh, etc.) have traditionally dominated the field, on the other hand great attention is being paid to non-noble metals due to their abundance, lower cost, and often excellent catalytic behavior.^7–10^

Among various non-noble systems, nanostructured Ni-based catalysts have been the subject of interest due to their overall good catalytic performance.^11–14^ Nevertheless, despite the known advantages, these metallic species are susceptible to agglomeration driven by their high surface energy, which can reduce their catalytic activity as the resulting larger aggregates block the access to part of the active sites. Therefore, the choice of the support is critical in maintaining the dispersion and stability of active sites. Within this context, Ni supported on zirconia (ZrO_2_) emerged as an interesting catalytic system, combining the high catalytic potential of Ni with the thermal stability and oxygen vacancy tunability of zirconia.^11–16^ ZrO_2_ is also well-known for its robustness, resistance to sintering, and ability to store and release oxygen, making it a suitable support for active metal clusters.^17,18^ Ni nanostructures have been studied as potential catalysts in hydrogenation reactions, where the efficient activation of molecular hydrogen (H_2_) is a critical step. In turn, the cleavage of the H–H bond has overall lower kinetic barriers over Ni.^19^ Experimental and theoretical data indicate that Ni can activate H_2_ molecules to provide hydrogenating species, likely through hydrogen spillover (Scheme 1). However, the mechanistic aspects of spillover are not fully understood.^8,10,20–25^ In fact, H spillover is important because it offers a pathway to enhanced catalytic performance by expanding the active sites beyond the initial H_2_ dissociation site, allowing hydrogen atoms to migrate and participate in reactions across a wider area of the catalyst or support.^8,20–25^ Studies focused on H spillover demonstrate that H atom migration and utilization can be accelerated through precise chemical and structural tuning. For instance, introducing H_2_O molecules lowers the migration barrier in Pt-loaded metal–organic frameworks,^26^ while phase transformations in tungsten oxide strengthen the spillover effect improving H-oxidation activity and CO poisoning resistance.^27^ Furthermore, facet engineering on metal nanocatalysts dictates whether spilled H is effectively utilized for reactions like alkyne semihydrogenation^28^ and hydrogenation of quinolines.^29^ The development of multisite electrocatalysts has unlocked new pathways for reactions such as N_2_ reduction.^30^ Also, complex coordination environments can be tailored to create highly active sites.^31^ By leveraging these complex atomic interactions, ultrathin nanowires exhibit strong orbital coupling and a self-complementary effect, leading to a multifunctional electrocatalytic performance for both oxidation and reduction reactions across diverse pH environments.^32^ These studies demonstrate the multifaceted approaches to manipulating and leveraging H spillover for advanced catalyst design and an enhanced catalytic performance. In our case, studying the spillover mechanism may shed light on how Ni clusters and the oxide support interact under hydrogenation conditions and on how the support surface is reduced, that is, how oxygen vacancies form. Hence, the nature of the interaction of Ni with the support and the presence of vacancies can influence the hydrogenation activity.^15–17^ This behavior points to the relevance of strong metal–support interactions in tuning catalytic properties and offers a potential route for engineering more efficient hydrogenation catalysts through computational methods.^33,34^

**Scheme 1:**
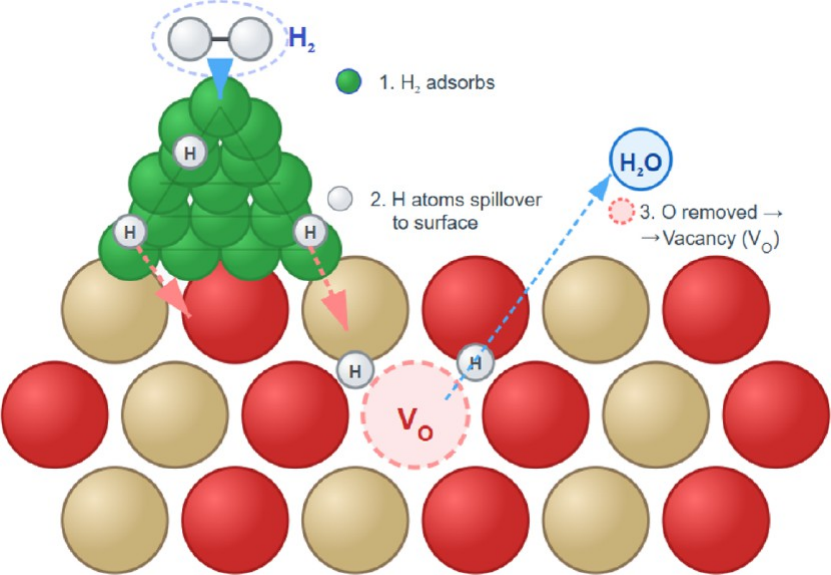
Illustration of O-Vacancy Formation via the H-Spillover Mechanism^a^

a Steps: (1) H_2_ adsorb and dissociate on the Ni cluster (green spheres) generating atomic H species; (2) the dissociated H atoms migrate onto the zirconia surface through spillover. Subsequently, surface O atoms (red) react with the H species to form H_2_O, which desorbs from the surface, resulting in the creation of oxygen vacancies (V_O_, dashed circle).

Thus, it is crucial to understand deeper the interaction of H_2_ with metal nanoparticles as well as how this molecule interacts with the support as it directly impacts the generation of surface oxygen vacancies. In fact, it is believed that the role played by the pair of electrons associated with a vacancy is important in hydrogenation reactions,^7,9,35,36^ and ZrO_2_-based supports have been investigated with such an objective.^37,38^ Overall, it is harder to generate vacancies on zirconia compared with other metal oxides,^39,40^ so that the catalytic activity is limited by a low density of vacancies In this sense, Ni nanoparticles are expected to modulate the surface behavior of ZrO_2_, possibly leading to a higher density of surface vacancies and, consequently, to improved catalytic properties. Clarifying the nature of this H_2_–Ni–ZrO_2_ interaction is essential for identifying the active sites at the atomic scale.

In this paper, we investigate by means of periodic density functional calculations the details of the interaction between H_2_ and a Ni cluster model deposited on ZrO_2_. We thoroughly examine how the H-spillover process takes place considering different H_2_ coverage and how the activated (H*) species react with lattice oxygen, eventually leading to the generation of a vacancy through H_2_O elimination.^8,10,20–25^ Importantly, the main goal of this study is not to simulate the catalytic behavior under operating conditions. Instead, the models aim to explore some of the fundamental aspects on the atomic level. While these models do not necessarily replicate actual supported Ni catalysts, experimental studies have demonstrated that low-dimensional Ni particles can be synthesized and stabilized on various oxide surfaces, being catalytically active.^41–43^ Therefore, the insights gained from this theoretical work are relevant to a broader field focused on low-dimensional, supported transition-metal catalysts.

## Computational Methodology

The computational investigation was conducted using supercells and pseudopotentials within the framework of the Quantum-Espresso open-source suite of computational codes.^44^ The PBE formulation of the generalized-gradient approximation was employed to account for quantum correlation and exchange contributions.^45^ Core–valence electron interactions were described using ultrasoft pseudopotentials with scalar relativistic correction, following the RRKJUS approach:^46^ Zr.pbe-spn-rrkjus_psl.1.0.0.UPF; O.pbe-n-rrkjus_psl.1.0.0.UPF; Ni.pbe-n-rrkjus_psl.0.1.UPF; H.pbe-rrkjus.UPF. Spin polarization effects were incorporated and the expansion of Kohn–Sham electronic valence states utilized plane-waves with energy thresholds of 50 Ry for kinetic energy and 400 Ry for charge density cutoff. Structural optimization was performed using the Broyden-Fletcher-Goldfarb-Shano (BFGS) algorithm integrated within the Espresso suite. Brillouin zone sampling was limited to the Γ-point due to the size of the system. Electronic occupations were treated with a Gaussian smearing of 0.01 Ry, while convergence criteria for self-consistency required forces on individual atoms below 1 × 10^–3^ Ry/Bohr and total energy variations between consecutive iterations on the order of 1 × 10^–4^ Ry.

The phase of the ZrO_2_ support (monoclinic, tetragonal, amorphous) is known to impact metal dispersion, metal–support interaction, and defect chemistry. Experiments indicate that the support phase may affect the Ni particle for catalysts prepared on different ZrO_2_ phases.^47,48^ In turn, methane decomposition studies indicate that Ni/amorphous ZrO_2_ yields smaller Ni particles and different coking behavior compared to monoclinic and tetragonal supports.^47^ In spite of these facts, monoclinic ZrO_2_ is the stable phase of pure zirconia up to approximately 1170 °C^49,50^ and therefore our model was built using this structure, in line with previous works.^51–53^ Among the low-index surfaces of monoclinic ZrO_2_, the supercell was structured to expose the (1̅11) plane, known as the thermodynamically most favorable face of zirconia;^54^ that makes it a realistic surface under catalytic conditions. The presence of Ni on the surface was taken into account according to a model presented by Pacchioni et al.,^55,56^ based on a stable ten-atom (Ni10) hemispherical geometry. This structure emulates a two-layered nanoparticle on zirconia (Ni10/ZrO_2_), in turn obtained as a section cut from a cuboctahedron shaped cluster consisting of a {7,3} configuration, i.e., seven atoms located in the bottom and the remaining three atoms in the top layer. The Ni10 cluster modeled here is smaller than the typical Ni particles reported in experiments.^57,58^ On the other hand, they serve as a representative model for the highly dispersed limit. These subnanometer clusters maximize the metal–support interface, capturing the low-coordination environments found at the steps and edges of larger experimental nanoparticles. The ZrO_2_ supercell model is illustrated in Figure S1. Atomic relaxation allowed free movement of the two uppermost (O–Zr–O) trilayers together with all Ni atoms, and eventually H_2_ molecules, without geometric constraints. Conversely, the bottom layer remained fixed at bulk positions (Figure S1). A vacuum region of at least 15 Å was established along the *z* axis to minimize unwanted interactions between periodic images.

The H_2_ adsorption energies were calculated as *E*_ads_ = (*E*_H_2_/Ni/ZrO_2__ – *E*_surf_ + *E*_H_2__)/*n*. In this formulation, *E*_H_2_/Ni/ZrO_2__ denotes the total energy of H_2_ adsorbed on the Ni10/ZrO_2_ system, while *E*_surf_ refers to the energy of the clean Ni10/ZrO_2_ systems; and *E*_H_2__ indicates the energy of *n* gas-phase H_2_ molecules. Negative *E*_ads_ values indicate energetically favorable (exothermic) adsorption.

The oxygen vacancy formation energy in the presence of H_2_ (*E*_vac‑H_) was calculated according to equation: *E*_vac‑H_ = *E*_H_2_/Ni/ZrO_2__ – (*E*_Ni/ZrO_2_–*x*_ + *E*_H_2_O(g)_), where *E*_H_2_/Ni/ZrO_2__, *E*_Ni/ZrO_2_–*x*_, and *E*_H_2_O_ represent the energy of the systems containing *n* adsorbed H_2_ molecules, those containing an oxygen vacancy, and a gas-phase water molecule, respectively. The total energy of gas-phase molecules was obtained by inserting them into a 25^3^ Å box. van der Waals interactions were incorporated using the DFT-D methodology developed by Grimme.^59^ The Climbing-image Nudged Elastic Band technique (CI-NEB)^60^ was utilized when required in order to determine energy barriers and transition states. The Bader method was used to partition the charge density and determine atomic charges.^61^ The DFT-optimized geometries, with corresponding total-energies, are provided as .xyz files in the Supporting Information Section.

In order to quantify the structural perturbation of the Ni10 cluster upon H_2_ adsorption, the root-mean-square deviation (RMSD) between the pre- and postadsorption geometries was computed, as detailed in Section S1 (Supporting Information). The observed deviations in Ni–Ni bond distances were evaluated within the framework of thermal displacement amplitudes and structural stability criteria. Previous studies have established that the critical RMSD for the onset of Ni melting corresponds to approximately 0.6 Å, representing ∼24% of the bulk interatomic distance of nickel (∼2.49 Å).^64^ Accordingly, we established a threshold based on the adsorbed (clean) cluster (Figure S1). For RMSD deviations below 0.2 Å upon H_2_ adsorption the system was considered essentially unperturbed, while deviations up to 0.6 Å indicate a moderate perturbation with some degree of geometric reorganization in the cluster, whereas for values larger than 0.6 Å we considered that the cluster was strongly perturbed. This metric enables systematic evaluation of the Ni10 cluster structural integrity under different hydrogen coverages.

## Results

From previous experimental and theoretical studies, it is accepted that the reduction of an oxide support usually initiates with the adsorption of H_2_ molecules on the supported metal particle, resulting in the formation of activated hydrogen species (H*), which may turn spillover to the support where they react with lattice O atoms, further generating vacancies via H_2_O elimination.^8,10,20–25^ In this sense, a deeper understanding of these steps is crucial to future improvements on the redox properties of oxide-based catalysts.^43,62^

### H_2_ on the Clean ZrO_2_ Surface

For comparison, we first investigate the H_2_ adsorption and vacancy formation on the clean stoichiometric zirconia (without Ni). In this case, a gas-phase H_2_ molecule was adsorbed considering several different initial positions; the calculated gas-phase H–H bond distance (*d*_H–H_ = 0.749 Å) is in good agreement with experiments (0.741 Å^63^). Our theoretical results indicate that the adsorption is overall associative (i.e., without spontaneous bond breaking) and only slightly exothermic. The adsorption energies vary from −0.07 to −0.14 eV (Figure 1) suggesting a weak interaction with the pure ZrO_2_ surface, pointing to a nonspontaneous cleavage of the H–H bond.

**Figure 1:**
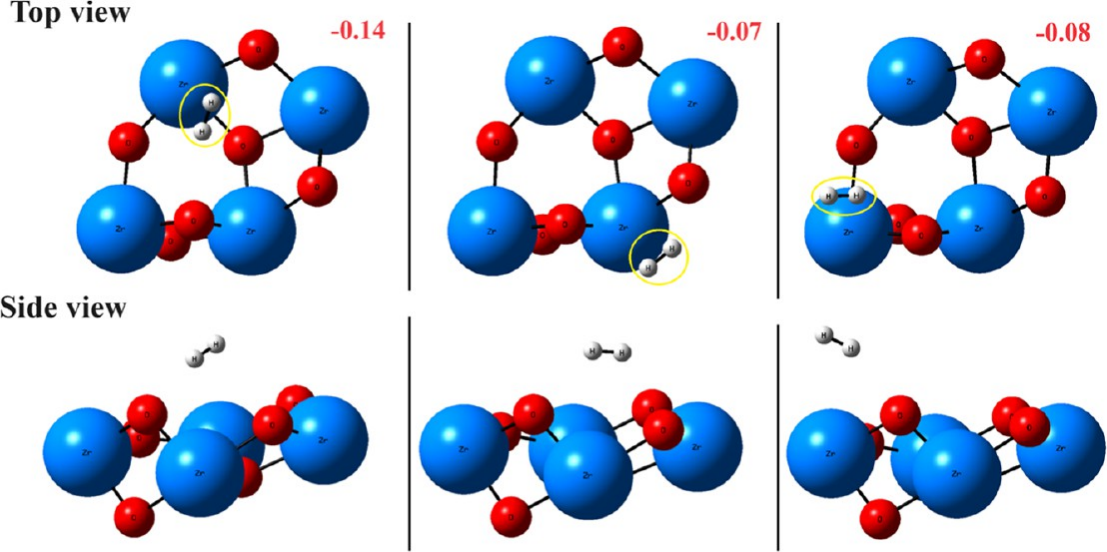
Illustration of H_2_ adsorption on the clean stoichiometric ZrO_2_ surface. Several different adsorption geometries were tested. The negative numbers in red represent exothermic adsorption energies in units of eV.

However, some dissociated configurations have been experimentally identified over zirconia in reducing environments.^64–66^ In principle, an homolytic dissociation provides either two hydroxyl (O–H) or two hydride (Zr–H) surface species, while an heterolytic dissociation generates both Zr–H and O–H species. It was proposed that Zr–H species are overall stable until ∼373 K while O–H species are stable above room temperature, suggesting that the presence of hydroxyls is favored at higher temperatures.^64,67^ After several attempts, our calculations indicate that a dissociative adsorption to form two separated Zr–H species is highly unfavorable energetically. In most cases at least one of the H migrates spontaneously to reform O–H and Zr–H bonds; in one case (Figure S2) the formation of two Zr–H was found very strongly endothermic, by +4.21 eV (note that the energy to dissociate a gas-phase H_2_ molecule is ∼+ 4.50 eV^68^). Thus, the formation of two separated Zr–H species from the H_2_ homolytic dissociation is very unlikely.

We establish here a threshold to consider whether an adsorbed H_2_ molecule is completely dissociated or not: whenever the H–H bond distances are larger than 1.00 Å the bond will be taken as completely dissociated; shorter distances will be considered either undissociated (those ∼0.75 Å) or “activated” (longer than 0.80 Å and shorter than 1.00 Å). Accordingly, Figure S3 depicts an heterolytic H_2_ dissociation, slightly endothermic (+0.24 eV) for vicinal Zr–H and O–H pairs (in this case, the H–H distance is 2.31 Å). Conversely, it is slightly exothermic (−0.10 eV) when H species are farther apart (*d*_H–H_ = 4.33 Å). The calculated heterolytic dissociation barriers of 0.53 eV for vicinal Zr–H\O–H and of 0.51 eV for the same species farther apart are relatively low and, in principle, suggest that H_2_ could be dissociated over stoichiometric regions of the zirconia surface, albeit with low thermodynamic stability. In fact, these results are in line with previous calculations reported in the literature^51,69,70^ as well as with experiments by Onishi et al.^64,67^ who assigned a sharp band around 1562 cm^–1^ due to Zr–H hydrides at ambient temperature. This band is relatively unstable, decreasing at temperatures higher than ambient, while bands relative to O–H stretching (3780 cm^–1^ and 3668 cm^–1^) increase, suggesting surface H migration from Zr to O surface sites under some higher temperature conditions. In fact, our calculations show that the homolytic cleavage of H_2_ to generate O–H species are overall endothermic with energies varying from +1.87 eV to +2.51 eV (Figure S4).

Assuming the presence of dissociated H* species on the surface, an O vacancy could be generated from a subsequent elimination of a H_2_O molecule. As previous studies identified a 2-fold coordinated lattice oxygen (O_2c_) as the most labile on the (1̅11) surface,^52,53^ this will be henceforth considered as the reference O atom regarding the surface vacancy formation.

The elimination of an H_2_O molecule leaves a vacancy with two electrons behind, where a vicinal Zr^4+^ may capture one of them to form a Zr^3+^ species. Experimentally, this is observed by reducing zirconia under H_2_ flow and relatively high temperatures.^64,66,67^ The existence of surface O vacancies can be confirmed by EPR spectroscopy, through a sharp peak at *g* = 2.003 attributed to F centers.^71^ Considering the H_2_/ZrO_2_ system, the elimination of H_2_O was found to be endothermic by +3.50 eV. So, the release of H_2_O was considerably less endothermic when compared with the direct O_2_ elimination (+5.93 eV).^52^ Previous investigation employing GGA+U calculations^40^ has also identified the O_2c_ vacancy as the most stable at the (1̅11) surface,^40^ but while the +U correction introduces quantitative shifts in energetics, it does not alter the relative trends across the different systems. Since our focus remains on these trends and relative energy differences, the standard DFT approach is qualitatively reliable and computationally efficient, justifying its use in this study.

As expected, once the vacancy is formed on the pure ZrO_2_, an eventual H_2_ adsorption nearby the hole will be much stronger if compared to the stoichiometric surface. As can be seen in Figure S5, this process is considerably exothermic (−1.66 eV), leading to the rupture of the H–H bond; one of the H* species occupies the vacancy, while the other forms a Zr–H hydride creating a highly stable system. Other configurations that form surface hydroxyls are energetically less stable (Figure S6).

Although dissociated hydrogen species eventually exist on the ZrO_2_ surfaces, their energetic stability is relatively low. Besides, the strong Zr–O bonds of zirconia make it energetically difficult to form O vacancies. Thus, supported Ni metal particles might facilitate the creation of these vacancies. As discussed previously, an important path toward the reduction of zirconia is through formation of surface O–H groups and reduced Zr ions. In this process, Ni may play a fundamental role in promoting H_2_ dissociation and further spillover of hydrogen atoms from the Ni cluster to the support, providing activated H* species in a continuous way.^72,73^ Thus, the generation of both H* species and surface vacancies can be controlled by the presence of Ni.

With that in mind, we now investigate how H_2_ molecules interact with Ni, how H* species are formed, and how they spillover towards the ZrO_2_ support. We also evaluate the impact on the surface reducibility through H_2_O elimination. Previous theoretical works carried out for similar systems served as a basis for the present investigation.^69,74,75^

### H_2_ Adsorption Screening

#### *n*-H_2_ (*n* = 1; H/Ni Ratio of 0.2)

Several initial H_2_ adsorption geometries have been probed for *n* = 1H_2_, as shown in Figure 2. Interestingly, we found that placing a single H_2_ molecule directly onto a Ni atom overall maintains the integrity of the of the H–H bond, so that the geometry optimization procedure has only stretched the molecule from ∼0.75 Å (gas) to ∼0.88 Å (adsorbed). This adsorption was exothermic by −0.77 eV (see Figure 2, system #5) characterizing an “activated” molecular state.

**Figure 2:**
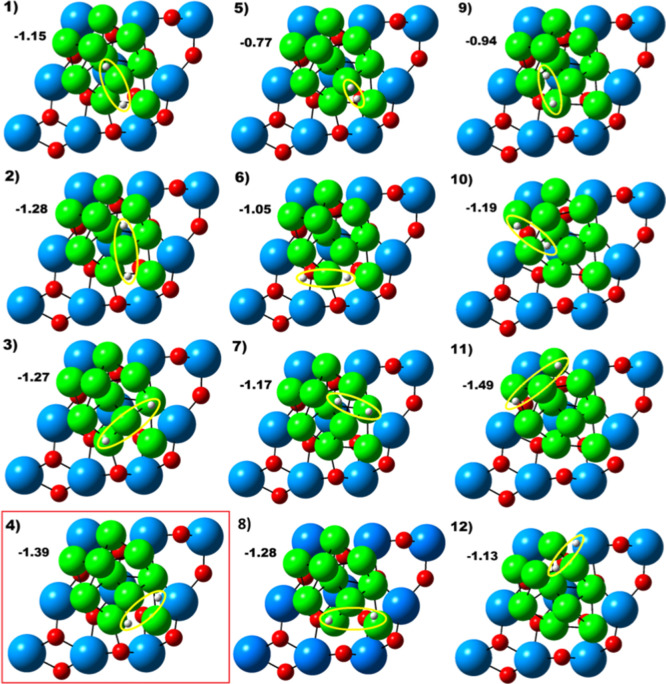
Top view of the optimized geometries (only the first layer of the zirconia surface is depicted) for single H_2_ adsorption on the supported Ni10 cluster. Several different positions were evaluated, as highlighted in yellow. The negative numbers in black represent exothermic adsorption energies in units of eV. The structure highlighted in red was used to probe the vacancy formation mechanism. Atomic color coding: Zr, blue; Ni, green; O, red.

Conversely, placing H_2_ onto interstitial regions (Ni–Ni bridge and 3-fold hollow sites, Figure S1) resulted in rather more favorable systems. In these cases, adsorption geometries are strong enough to cleave the H–H bond spontaneously (i.e., *d*_H–H_ > 1.00 Å). Such dissociative adsorption energies on Ni10 are exothermic, varying from −1.05 eV to −1.49 eV. As can be seen, the separated H* species remain attached to the cluster outer region in all cases (Figure 2).

Next, we investigated the spillover and vacancy formation processes, that is, the process in which H* species diffuse toward the support after surpassing some kinetic barriers and remove a lattice oxygen. To gain deeper insight, we chose the system in which H* is closer to the reference lattice O_2c_ atom (see Figure 2, system #4). The process starts with the H* diffusing from Ni10 to lattice O_2c_ to form a hydroxylic bond (O_2c_–H). An activation barrier of 1.44 eV was found for this step, which is endothermic (*E*_r_ = +1.07 eV); the full mechanism is depicted in Figure 3. Then, we considered a diffusion step of the remaining H* over the outer region of Ni10 and found a rather small activation barrier (*E*_a_ = 0.18 eV), suggesting that these species might be mobile on the outer region of the cluster. We finally investigated the formation of the H_2_O molecule (Figure 3) finding a relatively high a kinetic barrier (1.71 eV) with an endothermicity of +1.25 eV. Hence, the release of H_2_O with subsequent vacancy formation was found to be +3.26 eV up in energy compared to the reference initial state (Figure 2, system 4).

**Figure 3:**
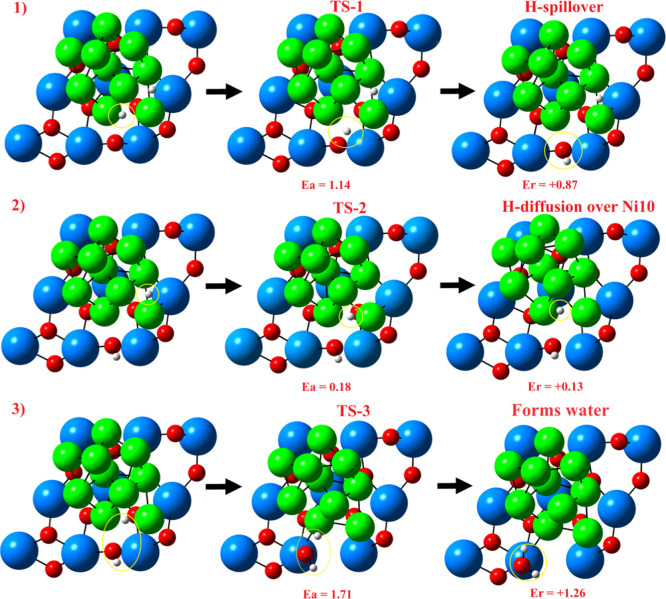
Illustration of the spillover (1), H* diffusion (2), and vacancy formation (3) steps considering the minimal H_2_ coverage (single molecule). The TS’s represent transition states. The numbers in red represent activation barriers (*E*_a_) and reaction energies (*E*_r_) in units of eV. The formation of H_2_O plus an O vacancy (step 3) is +3.26 eV up energy compared with the initial state (see Table 1). Atomic color coding: Zr, blue; Ni, green; O, red; H, white.

In principle, these results suggest that H* species from a single H_2_ might diffuse rather easily over the Ni10 outer region, although spillover and vacancy formation would be kinetically and energetically unfavorable. Based on the present results, it can be concluded that such a step is unlikely to take place considering a single H_2_ molecule (minimum coverage) . It is worth mentioning that the optimized geometry with dissociated H* species resulted in a slight perturbation of the Ni10 cluster (Table 2). Furthermore, electronic structure analysis based on the Bader approach^61^ reveals that the adsorbed H* species acquire some excess electron density, suggesting a net charge transfer from the Ni10 nanoparticle to the hydrogen adatoms (Table 2).

**Table 1:** Energetics of H_2_ Adsorption and Spillover Processes: *E*_ads_, Adsorption Energy; *E*_a(spill)_, Activation Barrier for the First H* Spillover Step; *E*_r(spill)_, Reaction Energy Related to the Spillover; *E*_vac_, Represents the Energy Penalty for Creating a Vacancy via H_2_O Elimination

N° of H_2_	*E*_ads_	*E*_a(spill)_ ^a^	*E*_r(spill)_ ^b^	*E*_vac_^3^
1	–1.32	1.44	+0.81	+3.26
2	–1.32	1.22	+0.70	+3.07
3	–1.33	1.17	+0.65	+2.97
4	–1.21	1.21	+0.63	+3.13
5	–1.17	1.17	+0.52	+2.93
6	–0.97	1.22	+0.50	+2.42
7	–0.96	0.91	+0.35	+2.53
8	–0.81	0.95	+0.30	+2.09
9	–0.87	1.04	+0.30	+2.00
10	–0.77	1.05	+0.07	+2.22
11	–0.67	0.89	–0.11	+1.30
12	–0.66	0.60	–0.51	+1.01

a Activation barrier for the 1st H* spillover step.

b Form O_2c_–H* species H_2_O removed leaving a vacancy behind.

All energies are in eV.

**Table 2:** RMSD Values Quantifying Structural Perturbation of the Ni10 Cluster upon H_2_ Adsorption, Calculated from Atomic Position Changes Relative to the Clean Cluster Geometry^c^

	^a^RMSD (Å)	Q (*e* ^–^)		
N° of H_2_ molecules	H_2_ adsorption	Ni10	ZrO_2_	^b^H
1	0.086	+0.620	–0.015	–0.605
2	0.157	+1.235	+0.003	–1.239
3	0.088	+1.885	+0.011	–1.897
4	0.114	+2.379	+0.118	–2.497
5	0.217	+2.686	+0.130	–2.816
6	0.203	+2.638	+0.198	–2.836
7	0.234	+2.673	+0.207	–2.880
8	0.344	+2.637	+0.230	–2.868
9	0.711	+2.783	+0.185	–2.968
10	0.64	+2.619	+0.098	–2.717
11	0.754	+2.510	+0.142	–2.652
12	0.753	+2.654	+0.117	–2.771

a Classification: RMSD <0.2 Å (considered unperturbed), 0.2–0.6 Å (moderately perturbed), >0.6 Å (highly perturbed).

b Sum of the charges for all of the adsorbed hydrogen atoms.

c Bader charges (Q­(*e* ^–^)) transferred between adsorbed hydrogen atoms (H), Ni10 cluster, and the ZrO_2_ support.

Subsequently, we conducted a large adsorption screening in which *n*-H_2_ molecules (*n* = 2–12) were progressively deposited on Ni10, resulting in an upper limit ratio of 2.4H atoms per Ni. The H_2_ molecules with gas-phase geometry were deposited preferentially onto hollow and bridged sites (Figure 4) and then allowed to relax without constraints. For all cases, we calculated the activation barrier of the first H* spillover step (Table 1). However, in order to alleviate the heavy computational burden associated with DFT calculations done to identify transition states, only the relative energy associated with the vacancy formation step (i.e., the second step) was calculated for the systems with *n*-H_2_ varying from 2 to 12 (Table 1).

**Figure 4:**
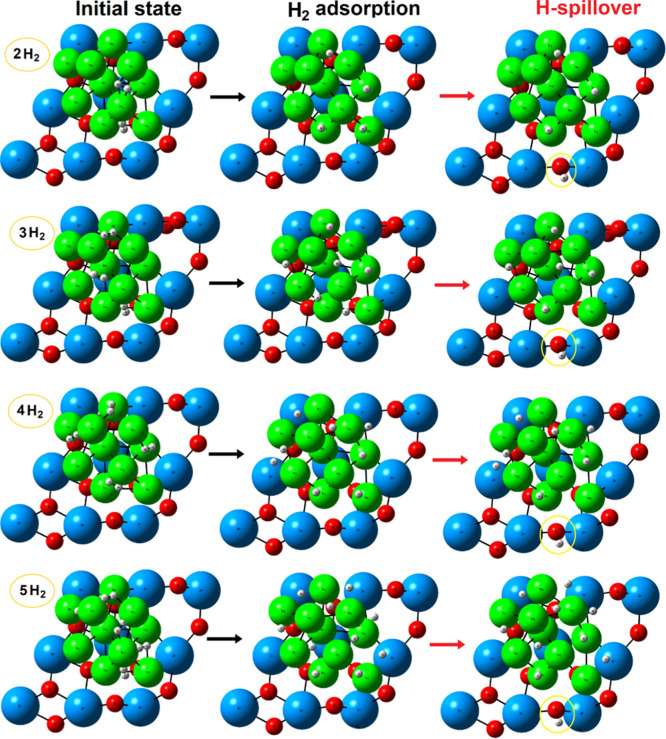
Initial, geometry-optimized and hydrogen spillover step for varying number of H_2_ molecules (*n* = 2–5) on the zirconia-supported Ni10 cluster. The corresponding energies (activation and reaction) are given in Table 1 and .xyz files are provided in the Supporting Information. Atom color coding: Zr, blue; Ni, green; O, red; H, white.

Within this approach, we aim to establish a plausible saturation range that could be expected under working (reducing) H_2_ atmospheres. Hence, the present calculations can be valuable for experimentalists at providing new insights regarding the complex processes during catalytic hydrogenation reactions. We emphasize that adsorption energies were normalized by the number *n* of H_2_ molecules.

#### *n*-H_2_ (*n* = 2–5)

To ensure consistency, we begin our screening study with the adsorption of two H_2_ molecules. For *n* = 2 (H/Ni ratio of 0.4) geometry optimization yielded an adsorption energy of −1.32 eV per H_2_, rather similar to the values obtained for a single H_2_ adsorption (Table 1). Both H–H bonds cleaved spontaneously, resulting in four individual H* species adsorbed on hollow and Ni–Ni bridge sites (Figure 4). The corresponding atomic distances between Ni and H are in the range ∼1.58–1.78 Å, characteristic of dissociative chemisorption; different initial geometries (not shown) overall generate similar results after geometry optimization. Further calculations revealed that the migration via spillover of an H* toward the reference O_2c_ lattice atom (Figure 4) is endothermic by +0.70 eV, with a barrier of 1.22 eV, and that the generation of a vacancy is +3.07 eV up in energy relative to the initial adsorbed state (Table 1).

When three H_2_ molecules are deposited on the Ni10 cluster (*n* = 3; H/Ni ratio of 0.6), all H–H bonds again dissociate spontaneously after optimization (Figure 4). The resulting H* species migrate to hollow and bridged sites, similar to those of the previous cases. The calculated adsorption energy remains comparable to the previous configurations (Table 1), reinforcing the notion that low H_2_ coverages are efficiently activated and stabilized on the Ni10 cluster. Spillover to the support remains energetically disfavored, although the associated endothermicity is slightly reduced to +0.54 eV. The corresponding kinetic barrier (Table 1) also indicates the limited feasibility of spontaneous H* transfer to the support under mild conditions.

For *n* = 4H_2_ (H/Ni ratio of 0.8), the same qualitative trends persist, i.e., all H_2_ molecules dissociate without energy barriers, with H* preferentially occupying hollow and bridge sites on the Ni10 cluster (Figure 4). The adsorption energy remains moderately exothermic (−1.21 eV, Table 1), albeit slightly less than that at lower coverages. Interestingly, the reaction energy for H* spillover (+0.63 eV) is similar to that of the previous case (+0.65 eV, *n* = 3), which did not suggest a shift in energetic driving forces as the cluster goes into a higher hydrogen coverage regime.

For the case where five H_2_ molecules are placed on Ni10 (*n* = 5; H/Ni ratio of 1.0), the system continues to exhibit behavior consistent with that of the previous coverages. All H–H bonds are spontaneously cleaved upon geometry optimization, hence leading to the formation of ten H* species distributed across hollow and bridge sites of the Ni10 cluster (Figure 4). The calculated adsorption energy is −1.17 eV per H_2_, therefore, only slightly less exothermic than the previous cases, suggesting a modest decline in adsorption strength as the hydrogen saturation increases. The H* species prefers to stay located on the outer region of the Ni10 cluster, suggesting that it is somehow resistant to hydrogen incorporation.

Bader charge analysis points to a charge redistribution for these systems (*n* = 2–5), confirming that the metal cluster becomes progressively more positively charged upon H_2_ dissociation (Table 2). This behavior highlights the electron-donating role of Ni in activating molecular hydrogen, so that dissociated H* species effectively become hydridic; typically, a smaller amount of charge is transferred from the ZrO_2_ support (Table 2). Thus, our calculations point to a trend of electron transfer from the Ni10 cluster to the adsorbed hydrogen atoms and from the support to the cluster. This charge redistribution confirms that the Ni cluster remains an effective electron donor at higher hydrogen loading.

Importantly, despite the progressive adsorption of H_2_ with complete dissociation of all H–H bonds, the Ni10 cluster geometry remained overall preserved for *n* = 2–4, as evidenced by RMSD values below 0.2 (Table 2). However, for *n* = 5, it increased, indicating a moderate perturbation within the cluster atomic structure. Despite this increase, our results indicate that so far the cluster can maintain a stable geometry under low H* coverages, without undergoing significant atomic rearrangement as it allows adsorption at hollow and bridge sites.

#### *n*-H_2_ (*n* = 6–9)

For the system containing six H_2_ molecules (*n* = 6; H/Ni ratio = 1.2), spontaneous dissociation of H–H bonds occurred for most molecules upon adsorption (i.e., *d*_H–H_ > 1.0 Å), while one H_2_ molecule remained in a partially dissociated (activated) molecular state (*H_2_) with an elongated H–H bond distance of 0.85 Å (Figure 5). Also, the formation of a terminal Ni–H hydride bond was observed; it is characterized by direct coordination of one hydrogen to a nickel atom with a bond length of 1.48 Å, shorter than H* species occupying hollow or bridging Ni–Ni sites. The calculated bond lengths are consistent with previously reported experimental^76^ and theoretical values.^77,78^ The presence of these intermediate adsorption states resulted in reduced energetic stability, as evidenced by a less exothermic H_2_ adsorption energy (−0.97 eV). The H* species preferentially occupy surface positions at the periphery of the Ni10 cluster, which retains its geometric structure with small differences compared to the *n* = 5 system (Table 2). Subsequent H-spillover process was found unfavorable under these conditions.

**Figure 5:**
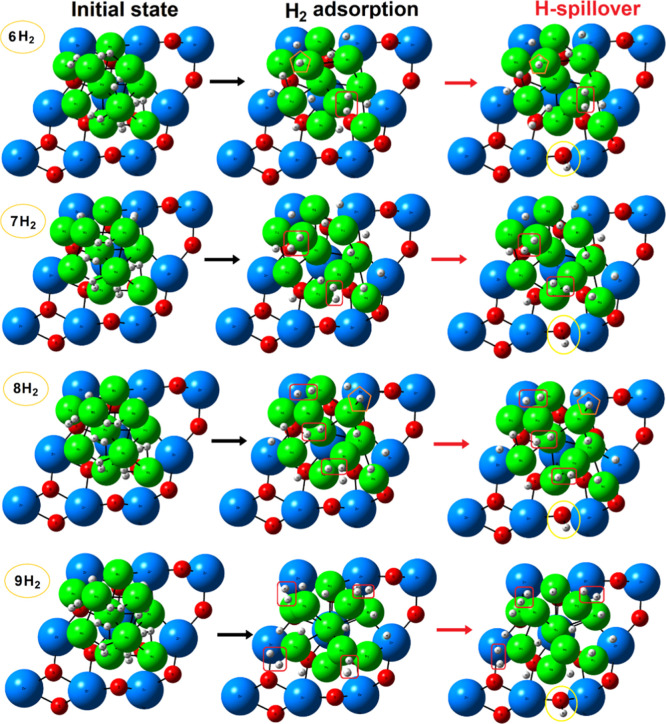
Initial, geometry-optimized and hydrogen spillover step for varying number of H_2_ molecules (*n* = 6–10). Table 1 gives the corresponding energies (activation and reaction). Red squares, orange pentagons, and purple hexagons highlight activated (adsorbed) *H_2_, hydride Ni–H bonds, and gas-phase H_2_, respectively. Atom color coding: Zr, blue; Ni, green; O, red; H, white.

As seen in Figure 5, a similar trend can be observed upon the adsorption of seven H_2_ molecules (*n* = 7; H/Ni ratio = 1.4), i.e., while the majority of H–H bonds are expected to spontaneously dissociate, two H_2_ molecules were found prefer an activated molecular state (*H_2_) with elongated H–H bond distances of ∼0.84 Å. The formation of a Ni–H bond was not observed in this case. The adsorption energy was similar to those of the previous case (−0.96 eV per H_2_), suggesting that the energetic cost of accommodating an additional H_2_ molecule is balanced by the formation of these weakly bound *H_2_ species. The Ni10 cluster exhibits a slightly greater perturbation, as evidenced by a broader distribution in Ni–Ni bond lengths (Table 2), reflecting increased strain due to higher H_2_ coverage. Interestingly, although the spillover process remains endothermic (+0.35 eV, Table 1), it is notably less endothermic than in the previous cases.

In the system containing eight H_2_ molecules (*n* = 8; H/Ni ratio of 1.6), the adsorption behavior becomes more complex with three H_2_ molecules remaining in an activated molecular form (*H_2_), illustrated in Figure 5. Also, a terminal hydride species (Ni–H) reappears, indicating the coexistence of multiple adsorption states for this H_2_ coverage. As expected, the remaining dissociated H* species spontaneously migrate to energetically favorable hollow and Ni–Ni bridged sites. This configuration contributes to a slightly less favorable adsorption energy (−0.81 eV per H_2_). The Ni10 cluster experiences a moderate structural perturbation (Table 2), with RMSD values indicating some geometric reorganization but maintaining an overall structural integrity. These changes reflect the accommodation of increased hydrogen amounts through subtle atomic rearrangements that preserve the geometry of the clusters.

The system containing nine H_2_ molecules (*n* = 9; H/Ni ratio of 1.8) indicates a shift regarding H_2_ adsorption, with four of them now retaining an activated molecular form (Figure 5). No additional terminal hydride species are formed at this coverage. Interestingly, the system exhibits a slightly more exothermic adsorption energy (−0.87 eV) compared to that of *n* = 8 (−0.81 eV). This behavior might be rationalized by examining the structural reorganization occurring; the RMSD increases (Table 2), indicating significant atomic rearrangement of the Ni cluster. This reorganization allows the cluster to adopt a geometry that better accommodates the nine H_2_ molecules, creating more energetically favorable interactions. Notably, the Ni10 cluster experiences substantially more pronounced structural perturbation, as evidenced by a sudden increase of RMSD (Table 2). This, in turn, indicates a significant atomic rearrangement necessary to accommodate the increased hydrogen loading. The structural deformation likely reflects the cluster’s response to minimize the steric repulsion. Interestingly, the adsorption energy becomes slightly more exothermic (−0.87 eV per H_2_) than the previous case (*n* = 8), suggesting that the structural reorganization facilitates more favorable hydrogen–cluster interactions.

For systems with *n* = 8–9, H-spillover remains unfavorable with endothermic energies and similar activation barriers compared to the *n* = 7 system (Table 1). This observation indicates that spillover kinetics exhibit relative insensitivity to coverage variations within this intermediate (*n* = 6–9; Ni ratios from 1.2 to 1.8) range. Bader analysis reveals a distinct charge redistribution (Table 2). For the *n* = 6–8 systems, the charge transferred from the Ni10 cluster remains relatively constant, indicating a consistent electron donation capacity despite varying hydrogen amounts. Concurrently, the charge transferred from ZrO_2_ exhibits a slight but gradual increase, reflecting an enhanced electronic interaction between the support and the Ni10 cluster. However, for the *n* = 9 system, the charge transferred from the ZrO_2_ support decreases while electron donation from the Ni10 cluster increases, suggesting that the structural perturbation observed at *n* = 9 weakens the electronic coupling between the cluster and the support.

#### *n*-H_2_ (*n* = 10–12): Exothermic Spillover

In the system with ten H_2_ molecules (*n* = 10; H/Ni ratio of 2.0), the adsorption reaches a critical threshold where five molecules remain in a partially activated molecular state, exhibiting stretched H–H bond lengths varying around 0.85–0.94 Å (Figure 6). Interestingly, one H_2_ molecule does not adsorb, preferring to remain in the gas phase near the Ni10 cluster. This was not observed in the previous systems. As a result, the structural perturbation of the Ni10 cluster slightly decreases due to the lack of full interaction with the gas-phase H_2_ molecule, alleviating some of the structural stress in the cluster (Table 2). The adsorption energy continues the trend of decreasing exothermicity (−0.77 eV per H_2_). Remarkably, the spillover energy drops significantly to only +0.07 eV, a substantial reduction compared to all prior systems.

**Figure 6:**
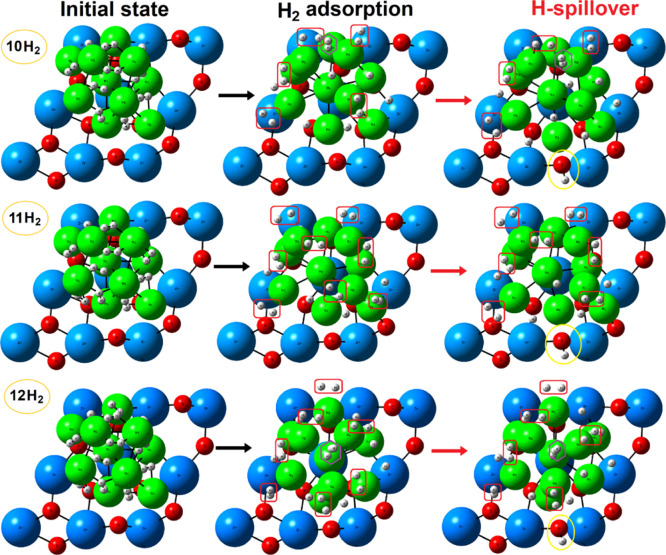
Initial, geometry-optimized and hydrogen spillover step for varying number of H_2_ molecules (*n* = 10–12). Table 1 gives the corresponding energies (activation and reaction). Red squares, orange pentagons, and purple hexagons highlight activated (adsorbed) *H_2_, hydride Ni–H bonds, and gas-phase H_2_, respectively. Atomic color coding: Zr, blue; Ni, green; O, red; H, white.

#### Exothermic Spillover

Upon increasing the number of adsorbed molecules to 11 H_2_ molecules (*n* = 11; H/Ni ratio of 2.2), as expected, part of the H–H bonds underwent spontaneous cleavage, populating available hollow and bridge sites on the Ni10 cluster; no further Ni–H hydrides are formed. However, a persistent presence of activated *H_2_ was observed (Figure 6) causing a more pronounced structural rearrangement of the cluster, as evidenced by a higher RMSD (Table 2). Importantly, the adsorption of 11 H_2_ molecules represents a saturation point in which the spillover process undergoes a key transformation: at this loading, the spillover process turns exothermic (Table 1). This indicates that the migration of H* species from the oversaturated Ni10 cluster to the support becomes energetically favorable.

This behavior can be attributed to high-energy hydrogen coverage, which creates a destabilized adsorbed configuration. The system is now sufficiently unstable so that migration to the support occurs. The overall adsorption energy drops to −0.67 eV, reflecting the destabilizing effects of this high hydrogen coverage. Thus, the spillover effectively relieves this destabilization by transferring excess hydrogen to the support. These results demonstrate that high hydrogen loadings alter the energetics and kinetics of the spillover process (Table 2), transitioning from an endothermic process at low coverages to an exothermic process at saturation conditions. We believe that this coverage-dependent spillover behavior could have important implications for the design of supported metal catalysts operating under high hydrogen pressures. However, such a study is beyond the scope of the present analysis.

Extending the hydrogen loading to 12H_2_ molecules (*n* = 12; H/Ni ratio of 2.4) confirmed the same trend observed for *n* = 11, with several activated *H_2_ and a similar level of structural perturbation in the Ni10 cluster (RMSD ≈ 0.75 Å). The adsorption energy remains moderately exothermic (−0.66 eV), consistent with the trend of decreasing stability at higher coverages. However, again one H_2_ prefer to stay in the gas phase, indicating that the cluster has finally reached a saturation point where further adsorption becomes unfavorable; a calculated system with 13H_2_ molecules (not shown) confirms this trend. Furthermore, the system exhibits a more exothermic and lower kinetic barrier associated with the spillover process (Table 1). These results suggest that the destabilization of the Ni10 cluster becomes so pronounced that hydrogen spillover to the support is favored. At this point the Ni10 cluster experiences maximum structural distortion under these conditions, with the geometric deformation somehow reaching a critical level facilitating the H migration process.

## Discussion

The present theoretical calculations revealed a coverage-dependent mechanism governing the hydrogen spillover and vacancy formation in Ni/ZrO_2_. The contrast between the pure ZrO_2_ surface and the Ni10 clusters highlights the role of metallic sites in nanostructures in facilitating H_2_ activation. While the clean zirconia surface (i.e., without Ni) exhibits only weak associative adsorption energies with a negligible tendency for H–H bond cleavage, the Ni10 cluster presents exothermic dissociative adsorption with spontaneous bond breaking at energy-favorable interstitial sites. The efficient dissociation suggests important ensemble effects where multiple Ni atoms cooperatively activate H_2_ molecules.

The most significant finding of this work was the systematic transformation of the spillover energetics as a function of the hydrogen coverage. At low coverages (H/Ni ratios of <2.0), spillover remains endothermic, preventing hydrogen migration to the support. This behavior aligns with the strong metal–hydrogen binding energies typically observed for transition metals, indicating why spillover is unfavorable at this point; overall, the dissociated species prefer to populate the outer region of the cluster. However, the progressive increase in the hydrogen coverage fundamentally alters this picture. Beyond H/Ni ratios of 2.2, spillover transitions to exothermic behavior with lowering of kinetic barriers (Table 1). This transformation can be understood by considering the nature of the interactions with the metal cluster, that is, high hydrogen coverages introduce significant hydrogen–hydrogen repulsive interactions on the metallic surface, effectively destabilizing the cluster and the dissociated species.

We show that H species does not migrate from the metal cluster to the oxide support at low surface coverage because it is energetically more stable to remain on the Ni cluster. This suggests that only at high partial pressures the H-spillover process becomes energetically favorable. At higher coverages, H_2_ adsorption energies on the Ni cluster gradually weakens and spillover becomes more favorable (Table 1). Experimental studies of hydrogen adsorption and desorption on Ni catalysts demonstrate that the equilibrium surface coverage depends on partial pressure. So, the highest hydrogen coverage considered in this work should be interpreted as a saturation condition. Temperature-programmed desorption studies on supported Ni catalysts show distinct desorption peaks for hydrogen between ∼375 and ∼560 K, reflecting the desorption of chemisorbed hydrogen from different types of Ni sites.^79^ Also, in line with our calculations, studies regarding the Au/TiO_2_ system indicate that spillover is directly related with the H_2_ partial pressure.^80^ Furthermore, hydrogen adsorption behavior and desorption temperature depend on coverage and surface site availability.^81^ Thus, metallic nanoparticles demonstrate the capacity to accommodate substantial hydrogen quantities prior to achieving saturation.^82,83^ These findings emphasize that higher hydrogen coverages are attained at elevated H_2_ partial pressures, so that the maximum coverage in our DFT provide insights into the energetic driving forces affecting spillover and vacancy formation.

The behavior exhibited in Figure 7 reveals the nature of the H_2_ coverage-dependent transformations. The adsorption energies point to a nearly linear decrease with increasing H_2_, indicating that the weakening of hydrogen-cluster interactions is likely due to lateral repulsion and site saturation effects (Figure 7a). This relationship also indicates that each additional H_2_ molecule contributes a consistent energetic penalty. The spillover activation barriers exhibit a somewhat less linear behavior (Figure 7b), although it is possible to identify a decrease from high values at low H_2_ coverage to lower barriers at saturation (∼*n* = 10). The spillover reaction energies also display linear behavior, transitioning from endothermic to exothermic regimes at a well-defined point (Figure 7c). In turn, the systematic reduction in O vacancy formation energies follows a similar linear trend that indicates elevated hydrogen coverages not only facilitate spillover but also enhance subsequent vacancy generation (Figure 7d). In principle, this suggests that vacancy formation could be tuned through hydrogen coverage control, optimizing catalyst performance under specific operating conditions.

**Figure 7:**
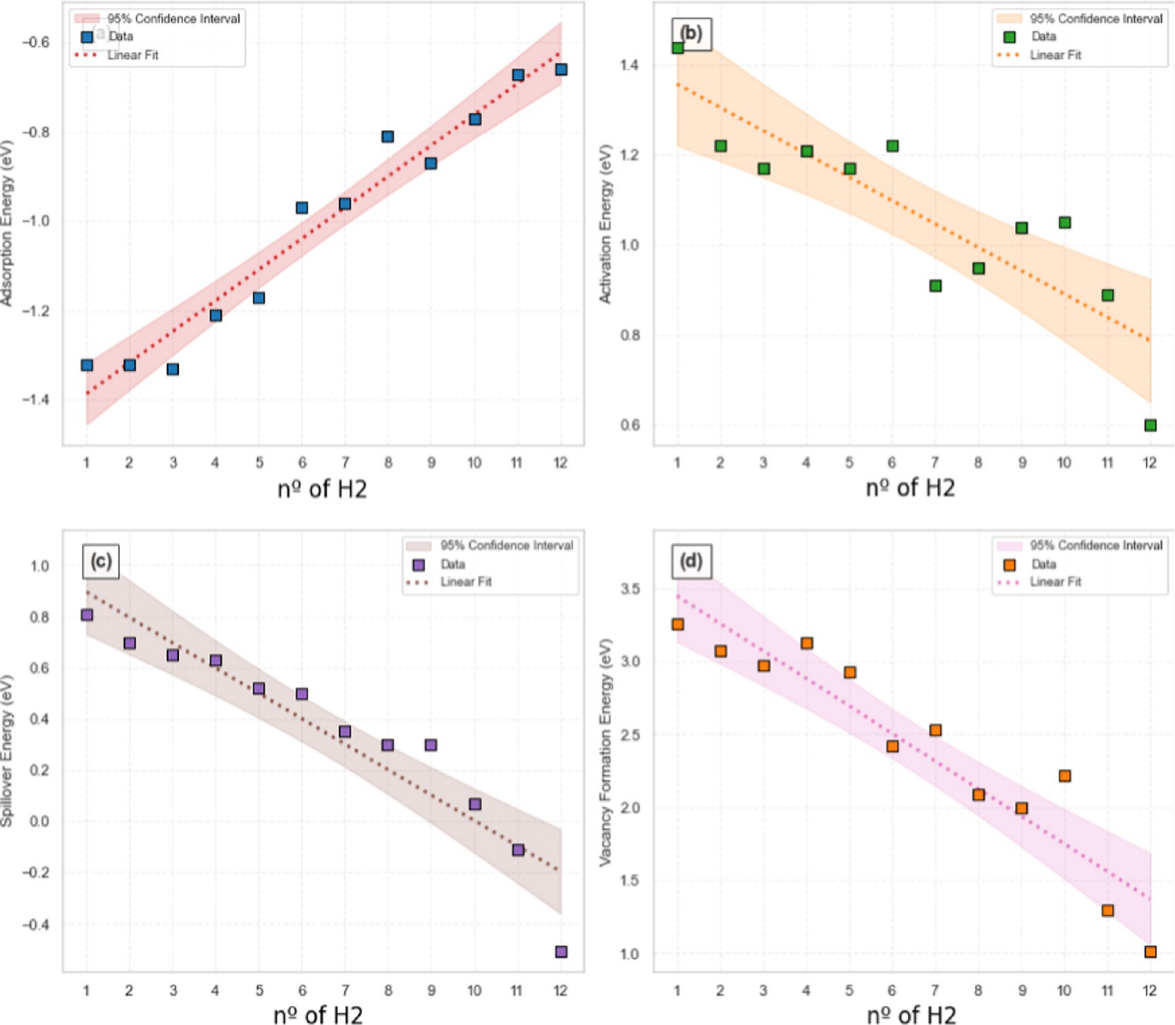
H_2_ coverage-dependent transformations on the Ni10 cluster. (a) Adsorption energy, (b) activation energy, (c) spillover energy, and (d) vacancy formation energy as a function of the number of adsorbed H_2_ molecules (1–12). The corresponding values are shown in Table 1. Data points are shown as colored squares. Shaded regions indicate 95% confidence intervals for the predicted values. Adsorption energy (a) shows a positive correlation (*R* ^2^ = 0.9548), indicating progressive stabilization; activation energy (b) decreases moderately (*R* ^2^ = 0.749); spillover energy (b) shows negative dependence (*R* ^2^ = 0.883); and vacancy formation energy (d) exhibits a decline (*R* ^2^ = 0.883).

The geometry of the Ni10 cluster upon H_2_ loading provides insights into the balance between H* accommodation and cluster stability. Figure 8a reveals that up to *n* = 8, the cluster presents some structural resilience. However, significant structural perturbation occurs at *n* = 9, indicating a threshold beyond which the cluster undergoes a more important degree of reorganization as it accommodates additional hydrogen atoms. The electronic properties (Figure 8b) reveal that Bader charge transfer increases up to *n* = 5 and then stabilizes at higher coverages. In turn, this behavior indicates that spillover is primarily governed by structural rearrangements of the metal particle rather than electronic charge transfer processes. The stabilization of charge transfer at intermediate coverages suggests that the electronic interaction between the cluster and support reaches equilibrium, while subsequent changes in spillover energetics are predominantly driven by cluster geometric factors, involving a reorganization of Ni atoms and lateral interactions among adsorbed hydrogen (H* and *H_2_) species.

**Figure 8:**
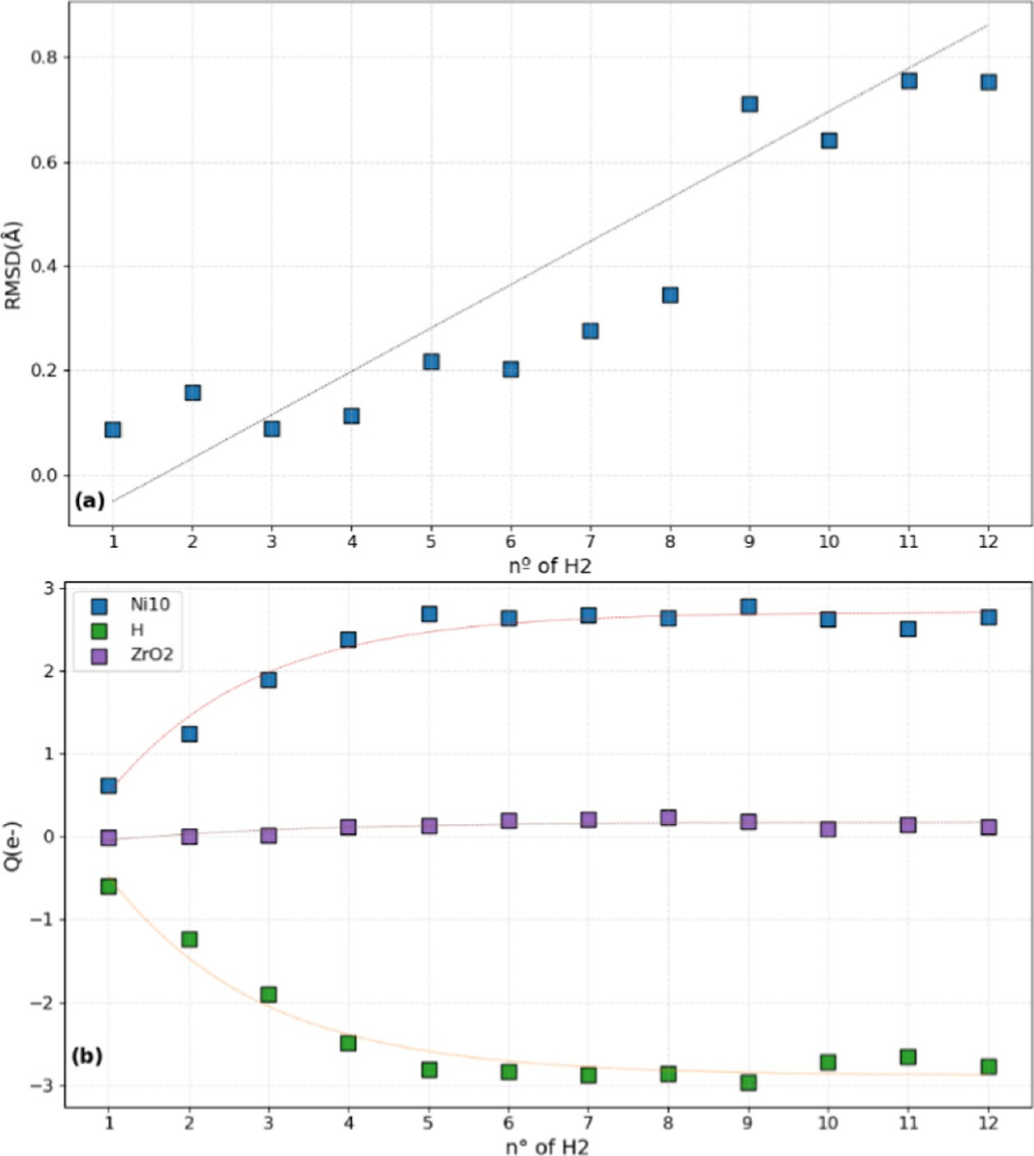
(a) Structural evolution of the Ni10 cluster via root-mean-square deviation (RMSD) under increasing H_2_ loading (*n* = 1–12). (b) Bader charge transfer to Ni10, hydrogen atoms (H), and to the support (ZrO_2_). The corresponding values are shown in Table 2.

By comparing our results with similar systems, we found that, e.g., Ru clusters over TiO_2_ spillover become thermodynamically favorable only when the metal cluster is saturated with ∼ 30H atoms, while on tetragonal ZrO_2_, spillover occurs at ∼ 24H atoms.^56^ This difference correlates with oxide reducibility; the O vacancy formation energies are substantially lower in TiO_2_ (∼3–4 eV) compared to ZrO_2_ (∼5–6 eV).^82,84^ Our results align with this trend, showing coverage-dependent spillover with a critical threshold at H/Ni > 2.0. In the case of Pt cluster, it was shown that it binds more strongly on TiO_2_ compared to graphene, resulting a marked difference.^74^ This suggests that stronger metal–support interactions might facilitate spillover. In our case, the stability of the Ni10 on monoclinic ZrO_2_ up to moderate coverages (cluster geometry preserved with minimal restructuring for H/Ni ≤ 1.6; Table 2) is in line with observed in other systems,^74,75^ highlighting how the specific metal–support influences both cluster integrity and spillover mechanisms during hydrogen adsorption.

The concept of hydrogen spillover is well established for some metal catalysts supported on ZrO_2_.^85–87^ However, obtaining detailed data for hydrogen spillover energy barriers is challenging. The literature often focuses on the overall reaction mechanisms and the role of the interface in activating H_2_ and CO_2_ rather than explicit coverage-dependent spillover. Our study on Ni/ZrO_2_ reveals a distinct coverage-dependent characteristic where the hydrogen spillover energetics transition from endothermic to exothermic with an increase in hydrogen coverage. Such behavior is likely driven by the geometric reorganization of the Ni cluster under high hydrogen loading, which lowers the energy barrier for H-migration to the support. This highlights the tunable nature of Ni/ZrO_2_ systems, where the extent of hydrogen loading plays a role in dictating the thermodynamics and kinetics of hydrogen spillover on the support.

## Conclusions

We presented a comprehensive theoretical investigation into hydrogen adsorption, spillover, and oxygen vacancy formation in a ZrO_2_-supported Ni nanoparticle model, namely, the Ni10 cluster (Figure S1). Some fundamental insights were revealed regarding the coverage-dependent nature of hydrogen spillover. For example, we showed that while the clean ZrO_2_ surface exhibits only weak, nondissociative H_2_ adsorption, the presence of Ni10 greatly facilitates spontaneous H–H bond cleavage. At low coverage, H_2_ dissociates efficiently on Ni10 inducing no significant structural perturbation. At this point, spillover remains endothermic and is kinetically hindered. Nevertheless, as coverage increases, structural reorganization of the cluster indicates the onset of hydrogen saturation. A transition occurs beyond *n* = 10H_2_ (i.e., H/Ni ratio >2), where the spillover process becomes energetically favorable. The calculations indicate that this shift is not driven by additional charge transfer, but instead it arises from increasing repulsion among adsorbed hydrogen atoms and geometric strain within the Ni10 cluster (Figure 8). The calculations revealed a point beyond which the cluster must reorganize its atomic structure in order to accommodate additional hydrogen (H* and *H_2_) species, eventually promoting spillover. Furthermore, our analysis indicates that higher hydrogen coverages not only facilitate hydrogen spillover but also promote the subsequent formation of oxygen vacancies on the ZrO_2_ support. In this sense, the progressive decrease in oxygen vacancy formation energy suggests that, in principle, hydrogen coverage could be leveraged to enhance the reducibility of the oxide support.

All in all, this study sheds more light on the microscopic mechanisms of hydrogen adsorption, spillover, and oxygen vacancy formation on oxide-supported metal nanoparticle catalysts. Our findings demonstrate that spillover and vacancy generation processes represent a complex, coverage-controlled phenomenon that requires careful consideration of structural, electronic, and energetic factors. The established relationships among coverage, energetics, and vacancy formation might provide a basis for tuning catalyst performance via H_2_ dosing strategies, particularly in systems where redox processes are important to the catalytic reaction.

## Supplementary Material







## Notes

Additional methodological details including a description of the structural analysis to quantify adsorption-induced distortions of the Ni cluster along with implementation aspects; and the supercell model, structural parameters, and adsorption configurations of H_2_ on both stoichiometric and defective surfaces (PDF)
The optimized geometries with corresponding total-energies (ZIP)
(PDF)
