# Supplementary material for: Coverage-Dependent Hydrogen Adsorption, Spillover, and Vacancy Formation in Ni/ZrO2 Systems: A First-Principles DFT Investigation
Source: ACS Omega. 2026 Apr 24;11(18):27143–58. doi: 10.1021/acsomega.6c00781 (PMC13177261; doi:10.1021/acsomega.6c00781)
Supplement: Supplementary file 1 [file ao6c00781_si_001.pdf]

SUPPORTING INFORMATION

Title: **Coverage-Dependent Hydrogen Adsorption, Spillover and Vacancy Formation in Ni/ZrO_2 systems: A First-Principles DFT Investigation**

Eugenio F. Souza*, Fábio S. Toniolo

Federal University of Rio Janeiro (UFRJ), Chemical Engineering Program of COPPE/UFRJ, CEP 21941-972, Rio de Janeiro, Brazil.

*Corresponding author: eugenio@peq.coppe.gov.br; eugenio_fs@yahoo.com

Eugenio F. Souza[1]

S.1 – Structural Analysis

To quantitatively assess the extent of structural perturbation experienced by the Ni_{10} clusters upon the adsorption of H_2 molecules, we employed the Kabsch algorithm [1,2] in conjunction with the root-mean-square deviation (RMSD) metric. This method enables a rigorous, rotation- and translation-invariant comparison between atomic configurations, providing a measure of the internal geometric rearrangements induced by adsorption.

The geometries of the Ni_{10} clusters were extracted from DFT optimizations (see the main text), both in their bare state (prior to adsorption) and after H_2 adsorption. To ensure that only intrinsic perturbations were considered, the analysis excluded any global translational or rotational contributions that could arise from cluster movement relative to the support.

The Cartesian coordinates of the Ni atoms in both the reference (pre-adsorption) and distorted (post-adsorption) structures were extracted from *.xyz* files (provided as SI). Let $\boldsymbol{P} = \{\overrightarrow{p_i}\}_{i=1}^{N} = 1N$ denote the atomic positions of the reference structure, and $\boldsymbol{Q} = \{\overrightarrow{q_i}\}_{i=1}^{N}$ the corresponding positions in the "perturbed" structure, where *N* is the number of Ni atoms. Both sets of coordinates were first translated such that their respective centroids coincided with the origin:

$$\overline{p_i^{\text{centered}}} = \overrightarrow{p_i} - \overrightarrow{\bar{p}}, \quad \overline{q_i^{\text{centered}}} = \overrightarrow{q_i} - \overrightarrow{\bar{q}}$$

where $\overrightarrow{p_i}$ and $\overrightarrow{q_i}$ are the centroids of sets $\boldsymbol{P}$ and $\boldsymbol{Q}$, respectively. To align the perturbed structure to the reference, we computed the optimal rotation matrix $\mathbf{R}$ that minimizes the RMSD between $\mathbf{P}$ and $\mathbf{Q}$ after alignment. This was achieved using the Kabsch algorithm [1,2], which proceeds as follows (1-5):

1. The covariance matrix $\boldsymbol{H}$ between the centered sets is computed as:

$$\boldsymbol{H} = \sum_{i=1}^{N} \left(\overrightarrow{p_i} - \overrightarrow{\bar{p}}\right)\left(\overrightarrow{q_i} - \overrightarrow{\bar{q}}\right)^T$$

2. Singular value decomposition (SVD) of $\boldsymbol{H}$ yields:

$$\boldsymbol{H} = \boldsymbol{U}\,\boldsymbol{\Sigma}\,\boldsymbol{V^T}$$

3. The optimal rotation matrix is given by:

$$\boldsymbol{R} = \boldsymbol{V}\,\boldsymbol{D}\,\boldsymbol{U^T}$$

where $\boldsymbol{D} = \text{diag}\big(1{,}1, \det(\boldsymbol{V}\boldsymbol{U^T})\big)$ ensures a proper rotation without reflection.

4. The aligned coordinates of the distorted structure were then computed as:

$$\overline{q_i}^{\text{aligned}} = \boldsymbol{R}\left(\overrightarrow{q_i} - \bar{\vec{q}}\right) + \bar{\vec{p}}$$

5. The RMSD between the aligned distorted structure and the reference was computed as:

$$\text{RMSD} = \sqrt{\frac{1}{N}\sum_{i=1}^{N} |\overrightarrow{p_i} - \overline{q_i}^{\text{aligned}}|^2}$$

This RMSD value serves as a quantitative descriptor of the internal structural distortion of the Ni10 cluster resulting from H_2 adsorption. A higher RMSD indicates greater deviation from the original geometry, reflecting larger internal rearrangements of the atoms due to interaction with adsorbed species.

Implementation – The entire procedure was implemented in Python using NumPy for linear algebra operations. Atomic coordinates were parsed from *.xyz* files generated from DFT calculations. The RMSD values reported in this work are accurate to four decimal places and have been verified to be independent of global rotations and translations. The code was provided as SI.

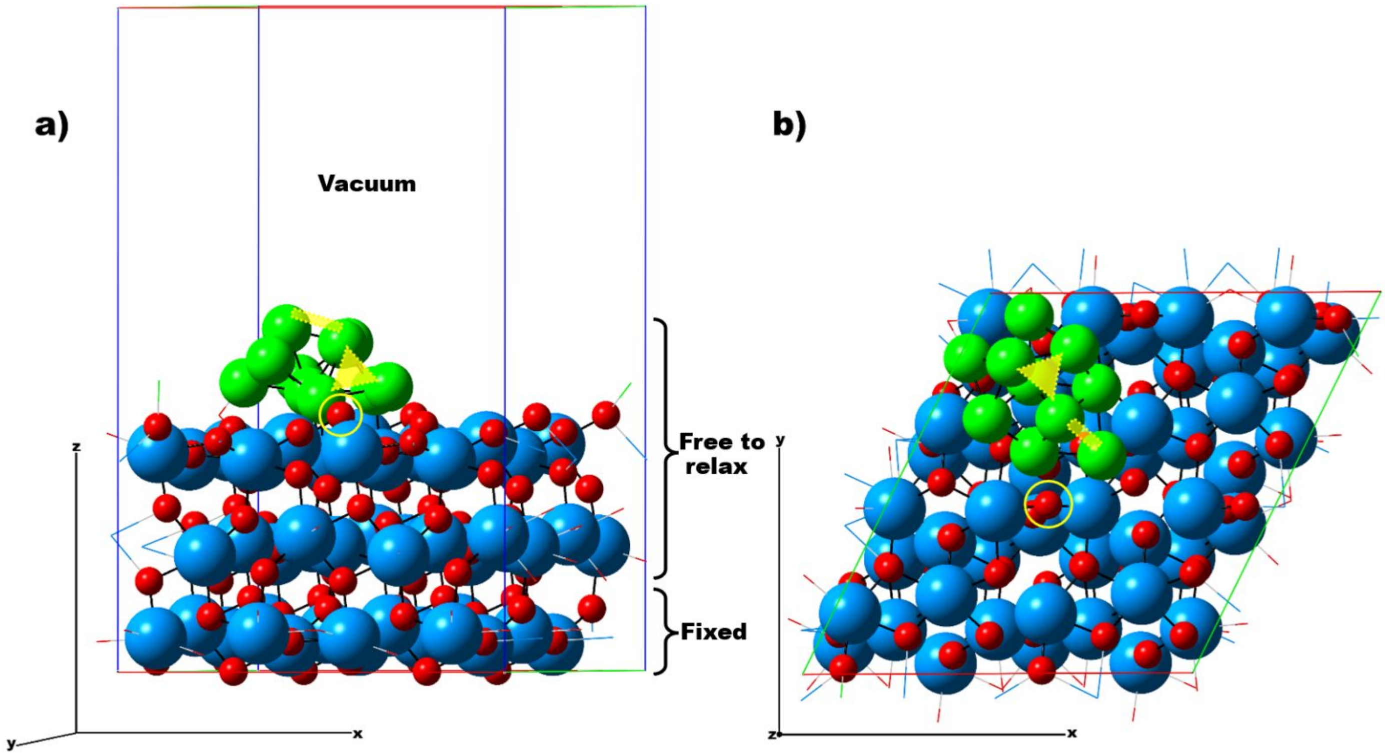

Figure S1 – Supercell built to model a Ni particle on the (-111) zirconia surface ($Ni_{10}@ZrO_2$); a) side view, b) top view. This model was employed to investigate multiple H_2 adsorption, spillover and O vacancy formation processes (see the main text). The average Ni-Ni bond distances in the adsorbed cluster was found to be 3.19 Å; the shortest one is 2.28Å while the longest is 5.73 Å. The yellow triangles and rectangles show some hollow and Ni-Ni bridges, respectively. The yellow circle highlights the reference lattice O_{2c} atom. Atom color coding: Zr, blue; O, red; Ni, green.

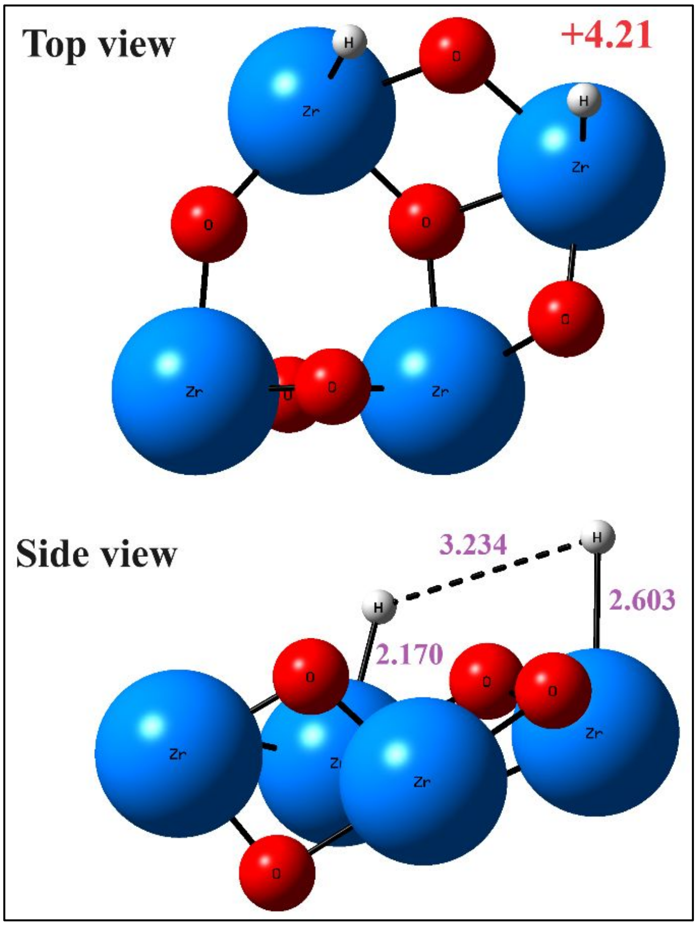

Figure S2 – Illustration of H_2 dissociative adsorption to form two Zr-H species over the stoichiometric ZrO_2 surface. The number in red represents the adsorption energy in units of eV. The purple numbers indicate bond lengths in Å.

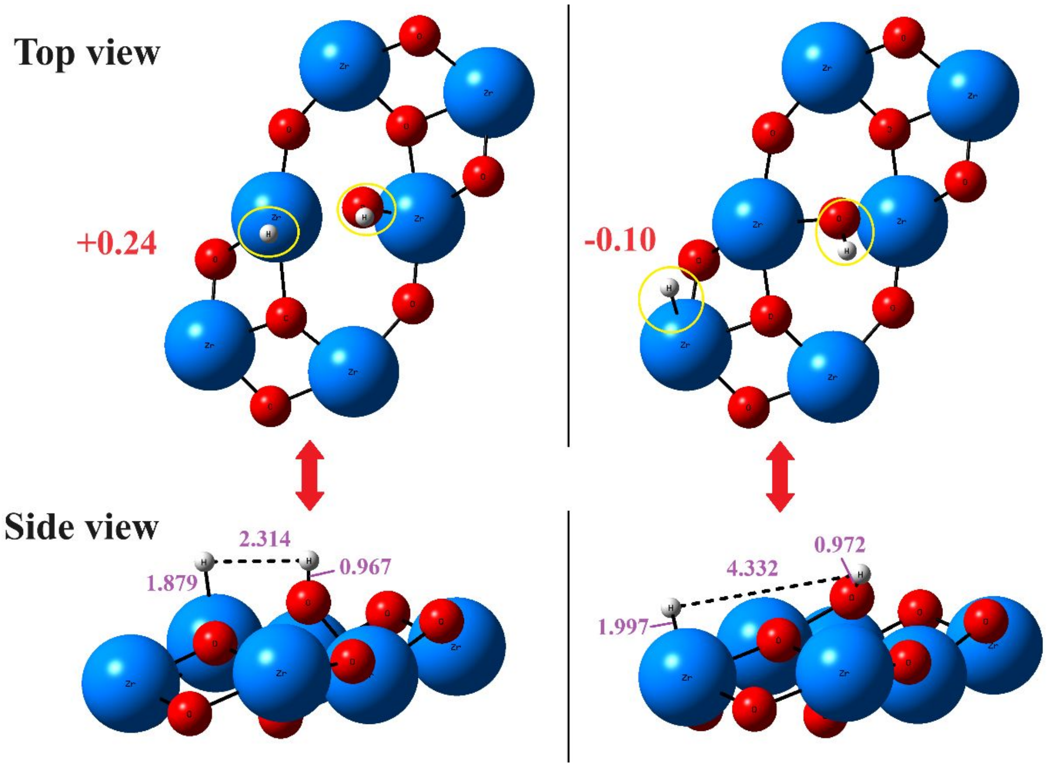

Figure S3 – Illustration of H_2 heterolytic adsorption to form O-H and Zr-H species over the stoichiometric ZrO_2 surface. The numbers in red represents the adsorption energy in units of eV; purple numbers indicate bond lengths in Å.

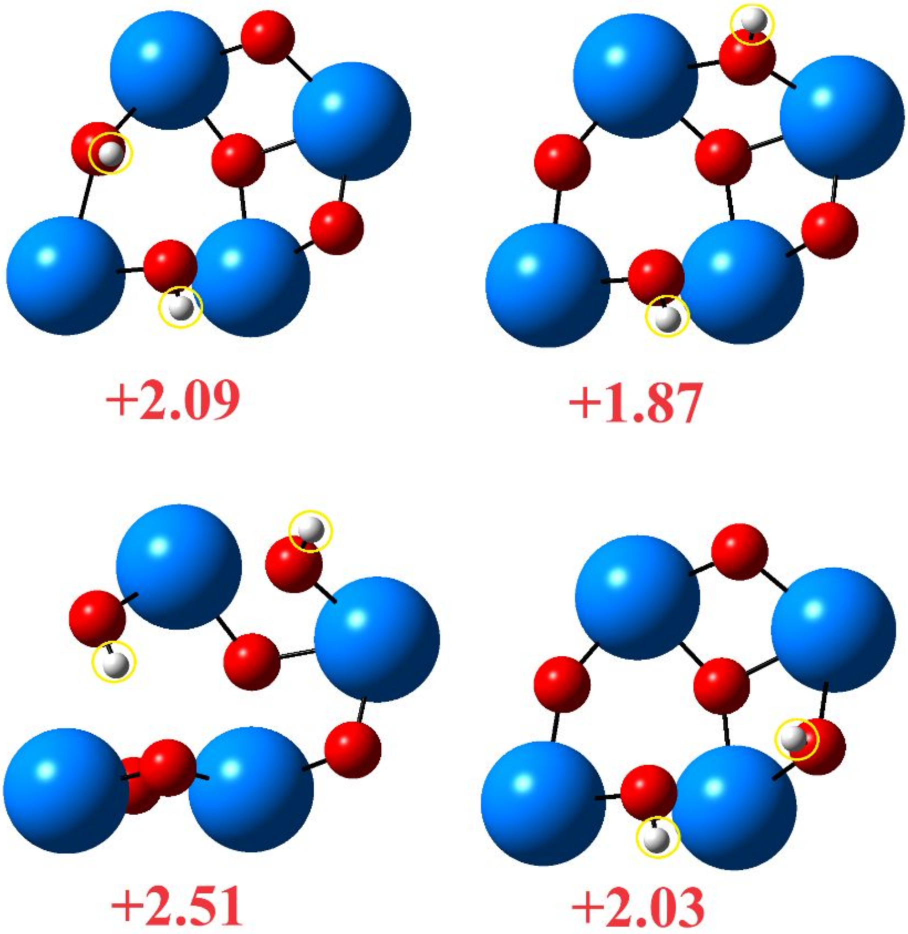

Figure S4 – Illustration of H_2 homolytic adsorption to form O-H species over the stoichiometric ZrO_2 surface. The numbers in red represents the adsorption energy in units of eV. Yellow circles highlight the O-H groups. Color coding: Zr, blue; O, red; H, white.

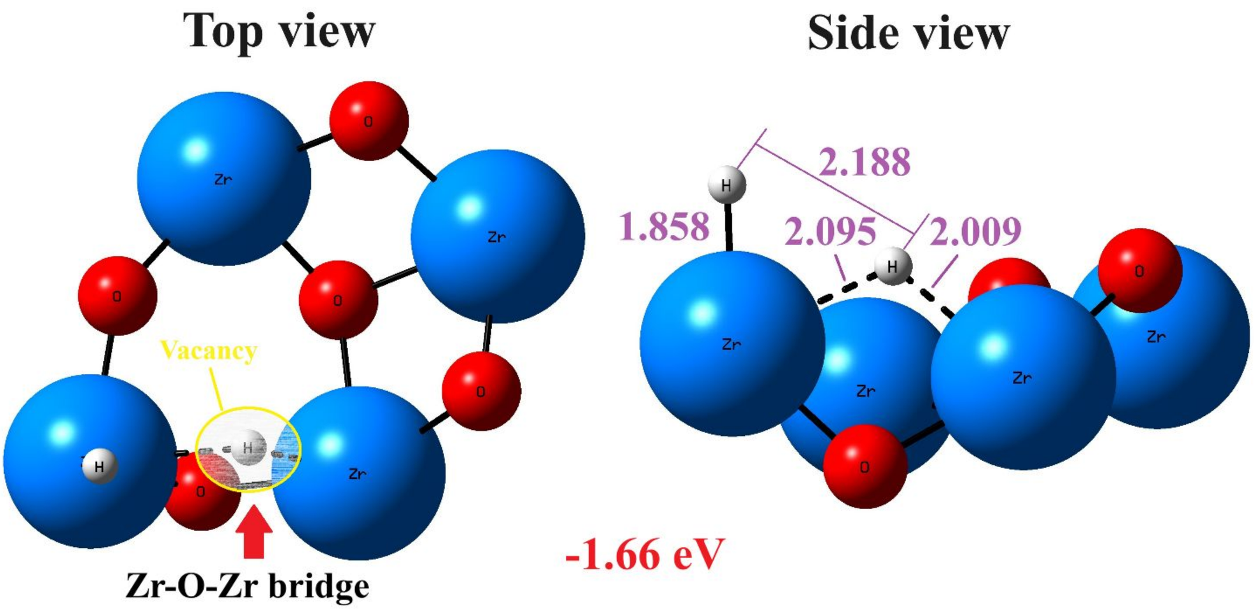

Figure S5 – Illustration of H_2 adsorption onto the vacancy, forming a Zr-H species over the oxygen defective ZrO_2 surface. The number in red represents the adsorption energy in units of eV, while the purple numbers indicate bond distances (Å). Yellow circle highlights the position of the vacancy.

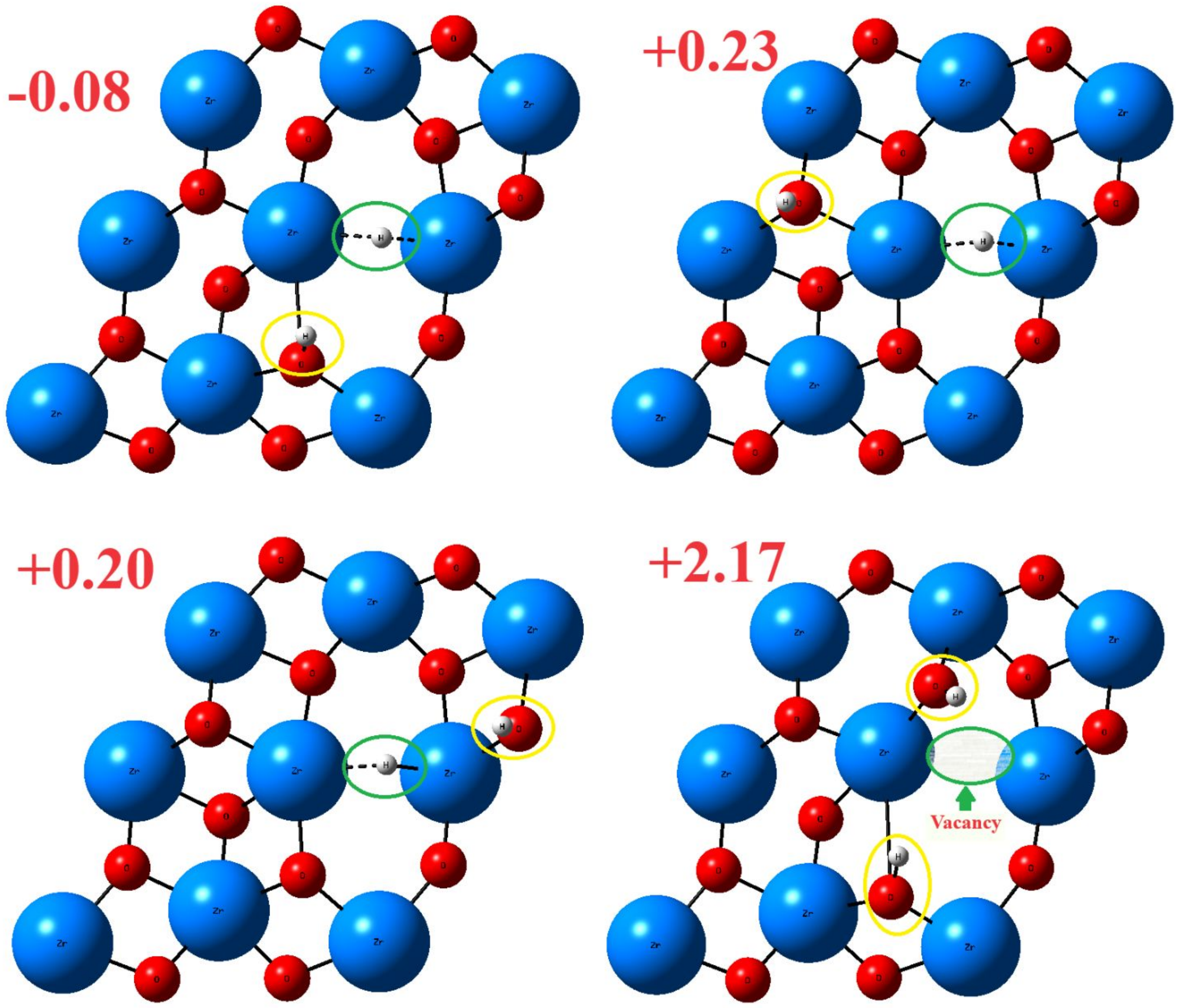

Figure S6 – H_2 adsorption onto the vacancy, forming a Zr-OH species over the defective ZrO_2 surface. The number in red represent the adsorption energies in units of eV. Yellow circle highlights the O-H species, while green circles indicate the vacancy position.

(1) Cezar, H. M.; Rondina, G. G.; Da Silva, J. L. F. Thermodynamic Properties of 55-Atom Pt-Based Nanoalloys: Phase Changes and Structural Effects on the Electronic Properties. *Journal of Chemical Physics* **2019**, *151* (20). https://doi.org/10.1063/1.5125689/197243.

(2) Kabsch, W. A Solution for the Best Rotation to Relate Two Sets of Vectors. *Acta Crystallographica Section A* **1976**, *32* (5), 922–923. https://doi.org/10.1107/S0567739476001873.
